# Presenteeism and Emotional Exhaustion as Mechanisms Linking Abusive Leadership to Non-Green Behavior in Hotel Enterprises: The Buffering Role of Co-Worker Support

**DOI:** 10.3390/ejihpe16030046

**Published:** 2026-03-23

**Authors:** Ahmed Mohamed Hasanein, Hazem Ahmed Khairy

**Affiliations:** 1 Management Department, College of Business Administration, King Faisal University, Al-Ahsaa 380, Saudi Arabia; 2 Hotel Management Department, Faculty of Tourism and Hotels, University of Sadat City, Sadat City 32897, Egypt

**Keywords:** abusive leadership, presenteeism, emotional exhaustion, non-green behavior, co-worker support, Conservation of Resources theory, Job Demands–Resources theory, hospitality industry, employee well-being, workplace sustainability

## Abstract

This study examines how abusive leadership influences non-green behavior among employees in five-star hotels in Egypt, drawing on Conservation of Resources (COR) theory and the Job Demands–Resources (JD–R) model. Using survey data collected from 400 full-time hotel employees, the study investigates the mediating roles of emotional exhaustion and presenteeism, as well as the moderating role of perceived co-worker support. Partial Least Squares Structural Equation Modeling (PLS-SEM) was employed to test the proposed relationships. The results indicate that abusive leadership increases emotional exhaustion and presenteeism, both of which contribute to higher levels of non-green behavior. Emotional exhaustion mediates the relationship between abusive leadership and non-green behavior, while presenteeism partially mediates the link between abusive leadership and emotional exhaustion. Additionally, perceived co-worker support buffers the negative effect of presenteeism on emotional exhaustion. By integrating COR and JD–R perspectives, this study advances understanding of the psychological mechanisms through which abusive leadership undermines environmentally responsible behavior. The findings offer practical insights for hospitality managers seeking to promote employee well-being and sustainability in high-pressure service environments.

## 1. Introduction

In the hospitality industry, leadership behaviors significantly shape employees’ psychological well-being, work attitudes, and sustainability-oriented behaviors (Jameel et al., 2025; Mahran et al., 2025). Among these, abusive leadership—subordinates’ perceptions of supervisors engaging in sustained hostile verbal and nonverbal behaviors, excluding physical contact (Tepper, 2000; Bhattacharjee & Sarkar, 2024; Hasanein & Elrayah, 2025)—has emerged as a critical concern due to its pervasive effects on employee functioning and organizational outcomes (W. M. E. Salama et al., 2025). In luxury hotels, where service excellence and emotional labor are highly demanded, such behaviors can deplete employees’ psychological resources, impair well-being, and contribute to counterproductive and environmentally detrimental behaviors that undermine sustainability initiatives (Lyu et al., 2016; Mackey et al., 2017; Ayad & Hasanein, 2024).

Recent surveys indicate that a substantial proportion of hospitality employees experience workplace stress and exposure to abusive leadership behaviors. For instance, studies report that up to 30–40% of hotel staff in high-pressure service environments encounter frequent verbal hostility or unfair treatment from supervisors, contributing to elevated levels of emotional exhaustion and presenteeism (Chia & Chu, 2017; Jameel et al., 2025). These findings underscore the prevalence and practical significance of toxic leadership in the hospitality sector, highlighting the need to examine its psychological and behavioral consequences, including impacts on employee well-being and sustainability-related behaviors.

Hospitality employees frequently face high emotional demands, requiring continuous regulation to maintain professional interactions with guests (Hasanein, 2024; de Oliveira & Sohn, 2025; Xiong et al., 2025). Exposure to abusive leadership can result in emotional exhaustion, defined as feeling emotionally drained due to chronic work stress (Maslach & Jackson, 1981; N. Yang, 2023). Emotional exhaustion, a key dimension of burnout, mediates the effects of negative leadership on employee performance and attitudes (Cropanzano et al., 2003). Exhausted employees may lack the motivation or resources to engage in pro-environmental behaviors, instead displaying non-green behaviors, such as disregarding environmental policies or engaging in wasteful practices (Paillé et al., 2014; H. Chen et al., 2024; Batarfi et al., 2025).

Presenteeism, attending work while physically or psychologically unwell, is another critical outcome (Koopman et al., 2002). In high-pressure service environments, employees may feel compelled to work while unwell due to fear of penalties or job insecurity (Yikilmaz & Surucu, 2025). Presenteeism exacerbates emotional exhaustion, as employees expend energy masking distress and maintaining productivity (Khairy, 2020), potentially reducing adherence to environmental norms (Biron et al., 2006; McLinton et al., 2023; Ayad et al., 2024). Thus, presenteeism may serve as a mediator linking abusive leadership and emotional exhaustion in hospitality contexts.

Co-worker support, defined as perceived help, empathy, and collaboration among colleagues, represents a vital social resource that mitigates workplace stressors (Khairuddin & Omar, 2016; Karasek et al., 1982). In five-star hotels, where teamwork is central to service delivery, supportive colleagues provide emotional reassurance, task assistance, and social belonging. Such support can buffer the detrimental effects of abusive supervision, reducing emotional exhaustion and preserving discretionary behaviors, including environmentally responsible actions (W. M. Salama et al., 2025; Santos et al., 2023).

The study is grounded in Conservation of Resources (COR) theory (Hobfoll, 1989) and Job Demands–Resources (JD-R) theory (Bakker & Demerouti, 2007). COR theory explains how individuals seek to acquire, protect, and conserve valued resources, such as energy, self-esteem, and social support, which are threatened under abusive supervision, leading to stress, exhaustion, and non-green behavior. JD-R theory posits that job demands, like abusive supervision, require ongoing psychological effort and may cause strain if resources are insufficient, whereas job resources, such as co-worker support, can mitigate these adverse effects.

Although research links abusive leadership to lower performance, job satisfaction, and higher turnover (F. Li et al., 2022; Singh et al., 2024), its impact on environmentally relevant behaviors remains underexplored. Supervisors communicate organizational priorities and shape norms; when they punish discretionary initiative, employees may withdraw from low-visibility tasks, such as energy saving, waste reduction, or reporting environmental noncompliance. Frontline employees’ everyday pro-environmental behaviors are crucial for hotel sustainability (Guerra-Lombardi et al., 2025). However, the literature connecting toxic leadership to non-green actions is sparse, highlighting a gap: interventions to improve service outcomes may not automatically protect green practices unless the underlying motivational pathways are specified and tested (Aboramadan et al., 2022; N. U. Khan et al., 2022; Zhou et al., 2022). Addressing this gap demonstrates that abusive leadership poses a latent threat to both service quality and environmental commitments.

The psychological mechanisms linking abusive leadership to non-green behavior—particularly through presenteeism and emotional exhaustion—require empirical investigation. Emotional exhaustion diminishes employees’ capacity to notice environmental cues, problem-solve, or engage in discretionary green tasks. Presenteeism shifts attention toward task survival rather than sustainability behaviors (S. Zhang et al., 2024; Dongmo & Tanova, 2025; Ng et al., 2025). While abusive supervision has been linked to exhaustion and counterproductive behaviors (I. A. Elshaer et al., 2025; Hasanein & Ayad, 2024; J. Li, 2025), few studies extend these outcomes to environmental behaviors. Empirically testing a model in which abusive leadership influences non-green behavior via presenteeism and emotional exhaustion advances understanding and supports targeted interventions to maintain sustainability under high job demands (W. M. E. Salama et al., 2025; J. Li, 2025).

Finally, co-worker support remains an underexplored moderator in sustainability-focused leadership research. JD-R theory suggests supportive peers provide emotional buffering, practical help, and normative reinforcement that sustain discretionary pro-environmental actions despite abusive supervision. Evidence shows co-worker support weakens the abusive supervision → emotional exhaustion link and reduces maladaptive reactions (H. Wang & Tang, 2022; Kursheed, 2023). Its role in maintaining green behaviors under abusive leadership, however, remains underexplored.

Accordingly, this study develops and tests a moderated mediation model in which abusive leadership influences non-green behavior through presenteeism and emotional exhaustion, while co-worker support mitigates these effects within the Egyptian hotel industry context. Grounded in COR and JD-R theories, the study contributes to the leadership and sustainability literature by integrating psychological mechanisms and contextual resources, and offers practical insights for hotel managers aiming to promote employee well-being and environmental responsibility in high-pressure service environments.

## 2. Literature Review and Hypothesis Development

### 2.1. Underpinning Theories

The current study integrates Conservation of Resources (COR) theory (Hobfoll, 1989) and Job Demands–Resources (JD-R) theory (Bakker & Demerouti, 2007) to explain the underlying psychological and behavioral mechanisms through which abusive leadership engenders non-green behavior among hotel employees. Specifically, it investigates how presenteeism and emotional exhaustion serve as key mediators in this process, and how co-worker support may buffer these negative effects.

According to COR theory, individuals strive to acquire, preserve, and protect valuable resources—such as energy, emotional stability, and supportive relationships—that enable them to cope effectively with workplace demands (Hobfoll et al., 2018). When resources are threatened or lost, individuals experience stress and engage in defensive behaviors to conserve what remains. Abusive leadership, characterized by sustained hostility and verbal aggression (Tepper, 2000), represents a potent resource-depleting stressor that drains employees’ emotional and psychological capital (Lyu et al., 2016; H. Chen et al., 2024; W. M. Salama et al., 2025). Such depletion can lead employees to experience emotional exhaustion and adopt maladaptive coping behaviors like presenteeism, which further accelerates their resource loss.

JD-R theory complements COR by conceptualizing the workplace as a system of job demands (aspects requiring sustained effort, such as abusive supervision) and job resources (factors that foster motivation and buffer stress, such as co-worker support) (Bakker & Demerouti, 2007). When job demands exceed available resources, employees experience strain and exhaustion, leading to impaired performance and disengagement from organizational goals (Schaufeli et al., 2009). Within the hospitality industry—known for its high emotional labor demands and guest-centric culture—these stress dynamics are particularly intense (Karatepe et al., 2014). Integrating both theories, the present model suggests that abusive leadership triggers a loss spiral of resources (COR) within a demand–strain–resource imbalance (JD-R), leading to presenteeism, emotional exhaustion, and ultimately non-green behavior.

### 2.2. Hypotheses Development

Abusive leadership, defined as sustained hostile verbal and nonverbal behaviors excluding physical contact (Tepper, 2000), represents a significant organizational stressor that undermines employee well-being. From the perspective of Conservation of Resources (COR) theory, abusive supervisors deplete employees’ valuable emotional and self-esteem resources, forcing them to expend additional energy on emotional regulation and impression management to preserve workplace harmony and job security (Hobfoll et al., 2018). This continuous loss of psychological resources ultimately manifests as emotional exhaustion—a central dimension of burnout—marked by feelings of emotional depletion, detachment, and reduced coping capacity (Y. Zhang & Liao, 2015; Maslach & Leiter, 2016). Empirical research within hospitality and service contexts consistently confirms this association, showing that abusive supervision is positively related to employees’ emotional exhaustion (Yu et al., 2023; Jasim et al., 2024). Employees exposed to such negative leadership often experience heightened psychological strain as they suppress negative emotions to maintain guest satisfaction, thereby intensifying emotional fatigue. Accordingly, the following hypothesis is proposed:

**H1.**  *Abusive leadership increases emotional exhaustion.*

Abusive leadership can also provoke presenteeism, defined as employees attending work despite illness or psychological distress (Koopman et al., 2002; Harris et al., 2007). Drawing on Job Demands–Resources (JD-R) theory, abusive supervision constitutes a demand overload that drains personal resources and elevates fear of managerial reprisal, thereby pressuring employees to remain at work even when unwell (Bakker & Demerouti, 2007). From a Conservation of Resources (COR) perspective, employees may engage in presenteeism as a protective strategy to prevent further resource loss, such as reputational damage or job insecurity, despite the cost to their health (Hobfoll, 1989). In hospitality settings, where frontline employees are highly visible and service demands are intense, individuals under abusive leaders may feel compelled to signal loyalty and compliance by attending work regardless of their well-being (W. M. Salama et al., 2025). This maladaptive coping behavior, however, further depletes limited energy reserves, increasing vulnerability to emotional exhaustion and other negative outcomes. Accordingly, the following hypothesis is proposed:

**H2.**  *Abusive leadership increases presenteeism.*

Presenteeism exacerbates emotional exhaustion because employees continue to expend effort even when their physical or psychological capacities are compromised. From the perspective of Conservation of Resources (COR) theory, this behavior initiates a resource loss spiral, in which already depleted employees overinvest their remaining energy to meet job demands, thereby accelerating burnout (Hobfoll et al., 2018). Empirical research consistently demonstrates that presenteeism is strongly associated with emotional exhaustion, impaired recovery, and overall burnout (Ren et al., 2024; Y. Zhang et al., 2025). In hospitality settings, the requirement for continuous emotional regulation—managing guest interactions and service expectations—magnifies the strain of working while unwell, further intensifying emotional fatigue (Lee et al., 2014; H. Khan et al., 2022). Based on these considerations, the following hypothesis is proposed:

**H3.**  *Presenteeism increases emotional exhaustion.*

Building on JD-R and COR principles, presenteeism serves as a key mechanism linking abusive leadership to emotional exhaustion. W. M. E. Salama et al. (2025) demonstrate that abusive leadership significantly increases employees’ presenteeism in hotel contexts. Employees subjected to hostile supervisory behaviors continue working despite psychological strain, which depletes their resources and reduces engagement with their jobs. This disengagement and ongoing emotional strain amplify the risk of emotional exhaustion over time. Thus, abusive leadership indirectly contributes to emotional exhaustion through its effect on presenteeism, highlighting presenteeism as a self-undermining behavior: a coping strategy intended to mitigate negative consequences of supervisory abuse but which ultimately intensifies employees’ emotional fatigue. Consequently, the following hypothesis is proposed:

**H4.**  *Presenteeism mediates the relationship between abusive leadership and emotional exhaustion.*

Perceived co-worker support—employees’ belief that colleagues provide emotional or instrumental assistance (Karasek et al., 1982)—serves as a vital job resource that replenishes depleted energy and fosters resilience. According to Job Demands–Resources (JD-R) theory, such resources buffer the negative effects of high job demands, helping employees maintain engagement and preventing burnout (Bakker & Demerouti, 2007). Socially supportive peers cultivate a sense of belonging and mutual understanding, which alleviates emotional strain and reduces the psychological burden of workplace stressors (Chiaburu & Harrison, 2008). In the hospitality industry, co-worker support has been shown to mitigate the adverse psychological impact of guest-related stressors and abusive leadership behaviors, preserving employee well-being (Karatepe, 2015; S. Xu et al., 2018). Therefore, when employees perceive strong co-worker support, their susceptibility to emotional exhaustion diminishes. Based on this rationale, the following hypothesis is proposed:

**H5.**  *Perceived co-worker support is negatively associated with emotional exhaustion.*

Beyond its direct benefits, co-worker support may also moderate the relationship between presenteeism and emotional exhaustion. From a Conservation of Resources (COR) perspective, supportive colleagues function as resource caravans, helping employees replenish depleted resources more effectively (Hobfoll et al., 2018). By providing emotional reassurance, sharing workloads, or offering practical assistance, co-workers can buffer the resource drain imposed by presenteeism. In team-oriented environments, these supportive interactions enhance morale and reduce stress, thereby weakening the impact of presenteeism on emotional exhaustion (J. W. Chen et al., 2021; Fahmy & Al Asrag, 2025). Conversely, when co-worker support is low, the emotional strain of working while unwell is intensified. Based on this reasoning, the following hypothesis is proposed:

**H6.**  *Perceived co-worker support moderates the relationship between presenteeism and emotional exhaustion, such that the relationship is weaker when co-worker support is high.*

Abusive leadership may also precipitate non-green behavior, defined as actions that harm or disregard environmental initiatives within the workplace (Paillé et al., 2019). From a COR perspective, employees subjected to hostility lose psychological resources such as moral engagement and self-control, making them less inclined to participate in pro-environmental behaviors (Hobfoll, 1989). Empirical studies show that destructive leadership diminishes organizational citizenship behaviors and promotes deviant or disengaged conduct (Tepper, 2000; Wu et al., 2018). In hospitality, employees who experience mistreatment may retaliate by ignoring green practices, wasting resources, or neglecting sustainability protocols (Zientara & Zamojska, 2018; Batarfi et al., 2025). Based on this reasoning, the following hypothesis is proposed:

**H7.**  *Abusive leadership increases non-green behavior.*

Emotionally exhausted employees tend to prioritize personal recovery over collective environmental objectives. From the perspective of Conservation of Resources (COR) theory, when individuals’ resources are depleted, they withdraw from discretionary behaviors that require additional effort, including engagement in sustainability initiatives (F. Wang et al., 2020; F. Xu et al., 2021). As a result, emotional exhaustion diminishes motivation to act pro-environmentally and increases the likelihood of counterproductive work behaviors. Empirical studies support this relationship, showing that emotional exhaustion undermines environmental citizenship behaviors while heightening eco-deviance (Paillé et al., 2019). In hotel settings, emotionally fatigued employees may neglect conservation rules, overconsume resources, or disengage from green initiatives. Accordingly, the following hypothesis is proposed:

**H8.**  *Emotional exhaustion increases non-green behavior.*

Finally, emotional exhaustion is expected to mediate the relationship between abusive leadership and non-green behavior. Abusive supervisors drain employees’ emotional resources, resulting in exhaustion and a diminished capacity or motivation to engage in sustainability practices. This process reflects a Conservation of Resources (COR) loss spiral, where ongoing resource depletion culminates in emotional collapse and environmentally detrimental behaviors (Hobfoll et al., 2018). Recent studies support this mediating role, showing that emotional exhaustion links destructive leadership to counterproductive and disengaged work behaviors (Ahmed et al., 2024; J. Li, 2025). In luxury hotel contexts, where sustainability outcomes rely heavily on employees’ routine actions and discretionary effort, this mediating mechanism is particularly salient. Based on this rationale, the following hypothesis is proposed:

**H9.**  *Emotional exhaustion mediates the relationship between abusive leadership and non-green behavior.*

**Figure 1:**
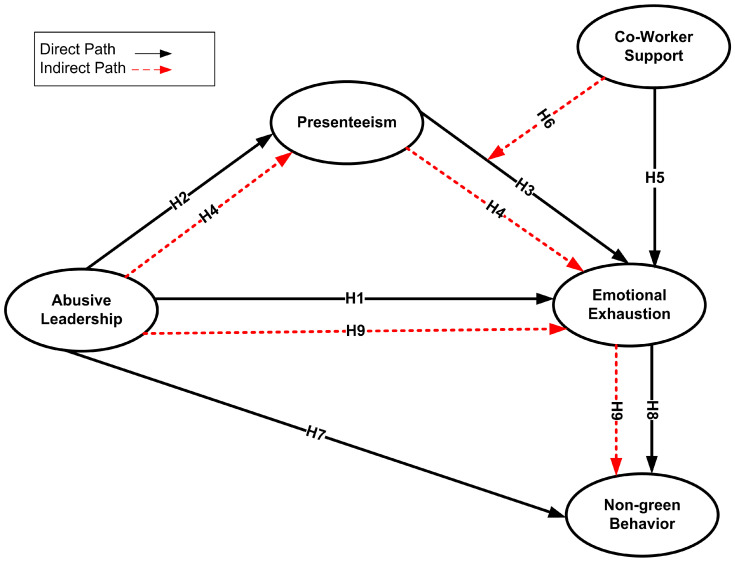
The theoretical framework of the study.

The theoretical framework of the study is illustrated below in Figure 1.

## 3. Methodology

### 3.1. Research Design

This study employed a quantitative, cross-sectional survey-based research design. Data were collected at a single point in time using a structured questionnaire to examine the relationships among abusive leadership, presenteeism, emotional exhaustion, perceived co-worker support, and non-green behavior among full-time employees working in five-star hotels in Egypt.

### 3.2. Measures and Instrument Development

This questionnaire comprising two primary sections. The first section addressed the study’s latent constructs and included 40 measurement items (see Appendix A Table A1). The second section gathered respondents’ demographic information, including gender, age, and educational background.

Abusive leadership was assessed using 15 items adapted from Tepper (2000). Employee presenteeism was measured with a six-item scale developed by Koopman et al. (2002). Emotional exhaustion was evaluated using a nine-item instrument originally developed by Maslach et al. (1986), while perceived co-worker support was measured through five items adapted from Karasek et al. (1982). Non-green behavior was captured using a five-item scale proposed by Paillé et al. (2019).

To ensure linguistic accuracy and cultural appropriateness, the English questionnaire was translated into Arabic by two bilingual experts and then back-translated into English following Brislin’s (1980) standard procedure. The translated version was reviewed by five academic specialists and ten hotel professionals, all of whom confirmed that no modifications were required. All constructs were measured on a five-point Likert scale ranging from 1 (“strongly disagree”) to 5 (“strongly agree”). Given the involvement of human participants, the research underwent formal ethical review and obtained approval from the authorized institutional ethics body, ensuring full compliance with the principles articulated in the Declaration of Helsinki concerning responsible conduct in research involving human subjects.

### 3.3. Sampling and Data Collection Procedures

Five-star hotels offer an ideal setting for exploring the interrelationships among abusive leadership, presenteeism, emotional exhaustion, and non-green behavior, given their intricate organizational structures and demanding service environments. Employees in such establishments often operate under significant pressure to maintain exceptional service quality, which increases their vulnerability to the detrimental consequences of abusive supervision. These adverse experiences can manifest as emotional exhaustion and presenteeism, where employees continue working despite experiencing psychological strain or distress.

Sustainability represents a fundamental aspect of luxury hotel operations, and even minor deviations from environmentally responsible behavior can considerably undermine a hotel’s overall environmental performance. Accordingly, examining these dynamics provides critical insights into how destructive leadership behaviors may impede an organization’s sustainability objectives. Moreover, the highly collaborative nature of work within five-star hotels underscores the potential role of co-worker support as a mitigating factor that can buffer the negative effects of abusive leadership on employee attitudes and behavioral outcomes.

Considering the geographic dispersion of Egypt’s hospitality sector and the scattered locations of five-star hotels, a convenience sampling technique was deemed the most appropriate. This non-probability method is commonly employed when probability sampling is constrained by logistical limitations, resource constraints, or the breadth of the target population (Cohen, 1988). Data were collected from full-time employees working in five-star hotels situated in the Greater Cairo Region between July and September 2025.

According to The Egyptian Ministry of Tourism and Antiquities (2022), the Greater Cairo area hosts approximately 30 five-star hotels. Prior to data collection, formal permission was secured from hotel management and human resources departments. Questionnaires were administered on-site to participating employees, with assurances of voluntary participation, anonymity, and confidentiality. Respondents were informed that all data would be analyzed in aggregate form only. A total of 500 questionnaires were distributed to hospitality employees, and 400 completed responses were received, resulting in a response rate of 80%. The 400 valid questionnaires obtained, satisfying the recommended minimum sample size criterion proposed by Hair et al. (2010)—specifically, a ratio of at least 10 respondents per observed variable (40 measurement item × 10 = 400). This ensured that the sample size was statistically adequate for subsequent analyses. Although the study followed the ‘10 responses per item’ rule, the final sample of 400 participants provides adequate statistical power for the PLS-SEM analysis, considering the number of latent constructs and hypothesized paths.

### 3.4. Data Analysis

Data were analyzed using Partial Least Squares Structural Equation Modeling (PLS-SEM) with WarpPLS version 8.0. PLS-SEM is a robust multivariate technique apt for complex theoretical frameworks in hospitality and strategic management research, effectively handling non-normal data distributions with small to medium-sized samples (Hair, 2014; Hair et al., 2017, 2019). The analysis followed a two-stage approach. First, the measurement model was evaluated by examining indicator loadings, internal consistency reliability (Cronbach’s alpha and composite reliability), convergent validity (average variance extracted), and discriminant validity using the Fornell–Larcker criterion and the heterotrait–monotrait (HTMT) ratio. Collinearity was assessed using variance inflation factors (VIFs).

Second, the structural model was assessed by estimating path coefficients, effect sizes (f^2^), coefficients of determination (R^2^), and model fit indices. Hypotheses were tested using bootstrapping procedures. Mediation effects were examined using the bootstrapping approach recommended by Preacher and Hayes (2008), while moderation effects were tested by incorporating interaction terms within the structural model. Statistical significance was evaluated using *p*-values and confidence intervals.

In addition, non-response bias was assessed using independent sample *t*-tests, revealing no significant differences (*p* > 0.05) between early and late respondents. To mitigate common method bias, participants were assured of anonymity, predictor and criterion variables were measured separately. Additionally, Harman’s single-factor test was conducted, indicating that no single factor accounted for the majority of variance, suggesting CMB is not a major concern.

### 3.5. Ethical Considerations

This study was conducted in accordance with established ethical research standards and the guidelines of the Declaration of Helsinki. Participation in the study was entirely voluntary, and all respondents were informed about the purpose of the research prior to data collection. Informed consent was obtained from all participants before they completed the questionnaire.

To ensure confidentiality and anonymity, no personally identifiable information was collected, and all responses were used solely for academic research purposes. Participants were informed of their right to withdraw from the study at any stage without any negative consequences. The study did not involve any procedures that could cause physical or psychological harm to the participants.

## 4. Results

### 4.1. Participants’ Profile

Table 1 presents the demographic and professional characteristics of the 400 participants included in the study. The sample is composed predominantly of male respondents, who make up about 78.12% of the total, while females account for 21.88%. Regarding age distribution, the largest group falls within the 30 to 45-year range (47.84%), followed by participants aged below 30 years (29.26%), and those older than 45 years (22.9%). In terms of educational attainment, most participants hold a bachelor’s degree (74.81%), while smaller proportions have completed high school (19.08%) or obtained a master’s or doctoral qualification (6.11%).

### 4.2. Descriptive Statistics of Study Variables

Table 2 presents the descriptive statistics for the study variables, including minimum and maximum values, mean scores, standard deviations, and distribution characteristics. Based on the 5-point Likert scale interpretation, abusive leadership has a mean value of 3.36, indicating a moderate level of perceived abusive leadership among respondents. The standard deviation (0.84) suggests a reasonable spread of responses. The negative skewness (−0.669) indicates that responses were slightly skewed toward higher values, while the kurtosis (−0.848) suggests a relatively flat distribution.

Presenteeism shows a mean score of 3.34, also reflecting a moderate level. Its standard deviation (1.03) indicates greater variability in responses compared to abusive leadership. The distribution is slightly negatively skewed (−0.483), and the kurtosis value (−0.979) suggests a flatter-than-normal distribution.

The mean score for emotional exhaustion is 3.58, which falls within the high level category. This indicates that respondents generally experienced higher emotional exhaustion. The standard deviation (0.94) reflects moderate variability. The skewness value (−1.120) shows a stronger clustering of responses toward higher scores, while the positive kurtosis (0.378) indicates a slightly peaked distribution.

Non-green behavior has a mean value of 2.80, representing a moderate level of such behavior. The standard deviation (1.09) suggests notable variation in respondents’ perceptions. The near-zero skewness (0.109) indicates an approximately symmetrical distribution, whereas the negative kurtosis (−1.193) reflects a flatter distribution.

Finally, perceived co-worker support reports a mean score of 3.18, indicating a moderate level of support among employees. The standard deviation (1.01) shows moderate dispersion of responses. The distribution is slightly negatively skewed (−0.304), and the kurtosis value (−0.794) suggests a relatively flat distribution.

Overall, the skewness and kurtosis values for all variables fall within acceptable ranges, indicating that the data are approximately normally distributed and suitable for further statistical analysis.

To facilitate interpretation of the descriptive statistics, mean values were categorized using equal-interval classification commonly applied to 5-point Likert-type scales in organizational and hospitality research. Following this approach, the scale range (1–5) was divided into five equal intervals: 1.00–1.80 (very low), 1.81–2.60 (low), 2.61–3.40 (moderate), 3.41–4.20 (high), and 4.21–5.00 (very high). These categories are descriptive rather than clinical, and no diagnostic or normative cut-off scores are implied. The classification is intended solely to enhance clarity and comparability of mean values across constructs.

### 4.3. Measurement Model

Table 3 summarizes the reliability and validity outcomes for the five main constructs used in the study. The table reports key measurement indicators, including factor loadings, Composite Reliability (CR), Cronbach’s Alpha (CA), Average Variance Extracted (AVE), and Variance Inflation Factor (VIF). All constructs show factor loadings above the acceptable threshold of 0.6, demonstrating strong indicator reliability. The CR and CA values for each construct exceed 0.87, indicating high internal consistency. Likewise, the AVE values range from 0.517 to 0.742, suggesting satisfactory convergent validity. The VIF values for all constructs are below 5, confirming that multicollinearity is not a concern. Overall, the results confirm that each construct in the model is reliable and valid, with items effectively measuring their respective theoretical dimensions.

Table 4 shows the relationships between the study’s main constructs. The diagonal values represent the square roots of the AVE, which are all higher than the correlations between constructs, confirming good discriminant validity. Overall, the findings suggest that the constructs are distinct but meaningfully connected, supporting the reliability of the measurement model.

Table 5 presents the Heterotrait–Monotrait ratio (HTMT) values used to assess discriminant validity among the study’s constructs. All HTMT values are below the recommended threshold of 0.85, indicating that each construct is distinct from the others. The results show moderate associations between AL and EE (0.673) and between AL and PCWS (0.656), while other relationships, such as between PR and EE (0.437) or between NGB and PCWS (0.498), are weaker. Overall, these values confirm that the constructs measure separate concepts and that discriminant validity is satisfactorily established within the model.

### 4.4. Model Fit

Appendix A Table A1 presents the assessment of model fit and quality using 15 indices recommended by Kock (2021). These indices evaluate the overall adequacy, predictive power, collinearity, and residuals of the structural model to ensure robust results. Key fit indicators show that the model performs well: the Average Path Coefficient (APC = 0.270), Average R-squared (ARS = 0.216), and Average Adjusted R-squared (AARS = 0.211) are all significant at *p* < 0.001, confirming meaningful relationships and explanatory power. Collinearity measures, including Average Block VIF (1.808) and Average Full Collinearity VIF (2.071), fall well below critical thresholds, indicating no multicollinearity issues.

Other indices, such as Tenenhaus GoF (0.385), Sympson’s Paradox Ratio (0.857), and Standardized Residuals (SRMR = 0.086, SMAR = 0.068), indicate good model fit. The Standardized Chi-squared (SChS = 20.252, *p* < 0.001) and threshold difference ratios (STDCR = 0.979, STDSR = 0.928) further support the model’s adequacy. Overall, all indices meet or exceed recommended criteria, confirming that the structural model is statistically sound, has strong predictive relevance, and is free from major estimation or multicollinearity issues.

### 4.5. Structural Model and Hypotheses Testing

**Figure 2:**
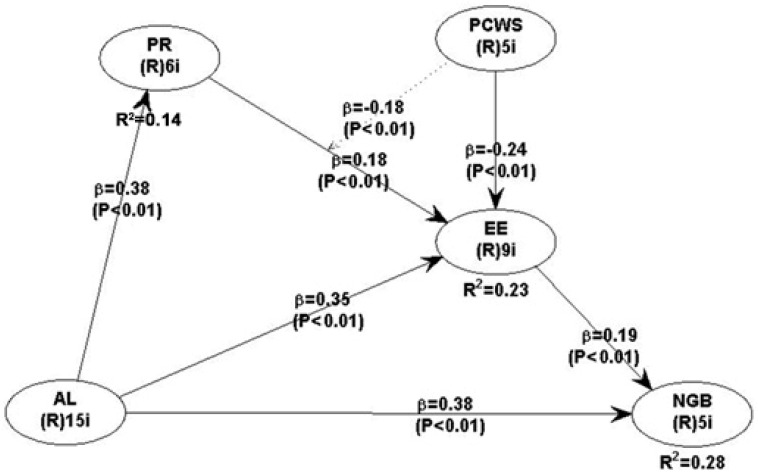
Final results of the study.

Table 6 and Table 7, and Figure 2 present the structural model results, including direct, indirect, and moderating effects among the study variables. All hypothesized paths (H1–H9) are statistically significant at *p* < 0.01, supporting the proposed relationships.

For direct effects, abusive leadership (AL) significantly increases Emotional Exhaustion (EE) (β = 0.35, f^2^ = 0.217) and Presenteeism (PR) (β = 0.38, f^2^ = 0.144), reflecting medium to large effects. In turn, PR contributes to higher EE (β = 0.18, f^2^ = 0.082), while perceived co-worker support (PCWS) mitigates EE (β = −0.24, f^2^ = 0.146), highlighting its protective role. Both AL (β = 0.38, f^2^ = 0.190) and EE (β = 0.19, f^2^ = 0.086) positively influence non-green behavior (NGB), though effect sizes are small to moderate. These results indicate that abusive leadership not only directly affects employee well-being and environmentally harmful behaviors but also operates through intermediary processes such as presenteeism and exhaustion.

**Figure 3:**
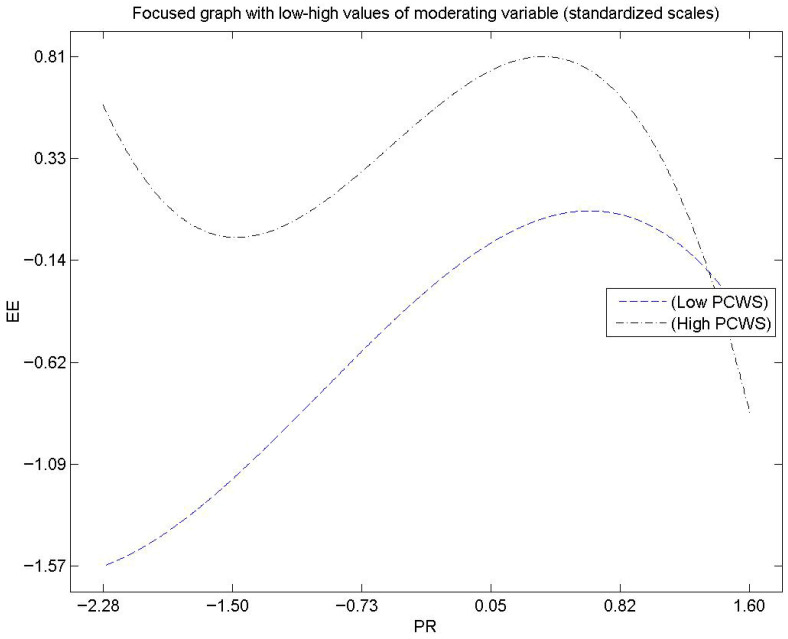
Moderating effect of PCWS.

The moderation analysis shows that PCWS weakens the impact of PR on EE (β = −0.18, f^2^ = 0.073), suggesting that supportive peers can reduce the emotional strain associated with working while unwell (see Figure 3). While the effect size is small, it underscores the practical value of fostering co-worker support in high-pressure hotel environments.

The model explains 14% of the variance in PR, 23% in EE, and 28% in NGB, indicating moderate explanatory power.

Table 7 summarizes the mediation results, tested using bootstrapping with the Preacher and Hayes (2008) approach. Presenteeism (PR) partially mediates the relationship between AL and EE (indirect effect = 0.068, SE = 0.032, t = 2.138, 95% CI [0.006, 0.131]), confirming that attending work while unwell contributes to emotional exhaustion. Similarly, EE partially mediates the effect of AL on non-green behavior (indirect effect = 0.067, SE = 0.029, t = 2.293, 95% CI [0.010, 0.123]), indicating that stress and burnout are key pathways through which abusive leadership undermines sustainability-related behaviors.

Overall, the results highlight that abusive leadership negatively affects employees both directly and indirectly, through presenteeism and emotional exhaustion, and that co-worker support can buffer some of these adverse outcomes. While some effects are modest in magnitude, the findings have practical relevance for interventions aimed at reducing stress, supporting employees, and promoting environmentally responsible behaviors in hospitality settings.

## 5. Discussion

The present study demonstrates that abusive leadership exerts significant psychological and behavioral effects on employees within hotel enterprises, providing empirical support for Conservation of Resources (COR) theory (Hobfoll, 1989) and Job Demands-Resources (JD-R) theory (Bakker & Demerouti, 2007). Consistent with COR theory, the positive relationship between abusive leadership and emotional exhaustion highlights how hostile managerial behaviors deplete employees’ emotional and cognitive resources, increasing susceptibility to fatigue and burnout (Akram et al., 2019). This finding aligns with prior hospitality research, where high service demands and frequent customer interactions exacerbate the stress associated with abusive supervision (Yu et al., 2020; W. M. Salama et al., 2025). In contrast, studies in broader service industries have reported weaker associations between leadership hostility and emotional exhaustion, suggesting that the intensity of customer-facing roles in luxury hotels may amplify these effects (Malik et al., 2023; Shin et al., 2021; Alsadaan, 2025). These differences underscore the contextual and cultural specificity of the hospitality sector, where hierarchical norms and customer expectations may heighten the consequences of abusive behaviors.

Abusive leadership also emerged as a significant predictor of presenteeism, indicating that employees under hostile supervision are more likely to attend work despite physical or psychological illness. This is consistent with previous hospitality studies linking managerial pressure and fear of job insecurity to presenteeism (W. M. Salama et al., 2025; S. Zhang et al., 2024; Yikilmaz & Surucu, 2025). These findings suggest that presenteeism should be understood as a coping strategy driven by resource depletion rather than simple behavioral noncompliance. Importantly, while the effect is statistically significant, the magnitude is moderate, indicating that other organizational and personal factors, such as workload allocation or individual resilience, may influence employees’ decisions to work while unwell.

The positive effect of presenteeism on emotional exhaustion corroborates prior evidence that attending work while impaired contributes to cumulative stress (Khairy, 2020; Khairy & Mahmoud, 2022; Silva-Costa et al., 2020; T. Yang et al., 2016). Although the effect size is relatively modest, it highlights the practical relevance of presenteeism as both an outcome of abusive leadership and a driver of further resource depletion. In hotel operations, where continuous service delivery pressures are pervasive, this finding emphasizes the importance of organizational policies addressing workload management and employee well-being (Arjona-Fuentes et al., 2019).

Perceived co-worker support (PCWS) demonstrated a protective effect, both directly reducing emotional exhaustion and moderating the impact of presenteeism on emotional exhaustion. These results are consistent with hospitality and tourism research emphasizing the buffering role of social support in high-stress service environments (Liu et al., 2023; Ersoy et al., 2023). From a COR perspective, supportive peer relationships help replenish depleted emotional resources, while JD-R theory frames PCWS as a job resource that mitigates the adverse effects of high demands. Notably, the moderating effect of PCWS, although small, suggests that peer support has limitations and may not fully offset the strain induced by abusive leadership. This indicates that interventions should combine peer support with broader organizational measures, such as leadership training and workload management, to maximize resilience and well-being (Agarwal et al., 2020; Durão et al., 2024; A. M. Elshaer et al., 2024).

Regarding discretionary workplace behaviors, abusive leadership directly increased non-green behavior, while emotional exhaustion also predicted higher non-green behavior. These findings extend the hospitality literature, which has primarily focused on customer service outcomes, by demonstrating that leadership-induced resource depletion can also undermine sustainability-oriented behaviors, aligning with the findings of Byrne et al. (2014), Boeske (2023), and W. M. E. Salama et al. (2025). Mediation analyses revealed that presenteeism partially mediates the effect of abusive leadership on emotional exhaustion, and emotional exhaustion mediates the relationship between abusive leadership and non-green behavior. This cascading mechanism highlights the critical role of psychological strain in translating leadership practices into environmentally counter-productive behaviors—a perspective that remains underexplored in hotel-specific sustainability research.

## 6. Theoretical Implications

This study makes several important contributions to theory by extending the understanding of how leadership behaviors affect employee well-being and organizational sustainability. First, it broadens the application of the Conservation of Resources (COR) theory (Hobfoll, 1989) by demonstrating that abusive leadership functions as a potent stressor that systematically depletes employees’ psychological and emotional resources. Previous COR-based research has largely emphasized how resource loss affects burnout, stress, and general well-being; the current findings highlight that resource depletion also extends to discretionary behaviors, particularly environmentally relevant practices. This suggests that employees under abusive supervision are not only emotionally strained but also less capable of engaging in proactive, pro-environmental behaviors, establishing a novel link between resource depletion and sustainability outcomes.

Second, the study provides a nuanced application of the Job Demands-Resources (JD-R) theory (Bakker & Demerouti, 2007) by showing that job demands, exemplified by abusive leadership and presenteeism, interact with job resources such as co-worker support to shape employee outcomes. The identification of presenteeism as a mediator between abusive leadership and emotional exhaustion illustrates the pathway through which resource loss manifests in strain-related behaviors. Simultaneously, co-worker support buffers this negative impact, offering empirical evidence that job resources can mitigate the harmful effects of excessive job demands. This integration underscores the importance of considering both the detrimental and protective factors in the work environment when studying stress and behavioral outcomes.

Finally, the research advances theoretical understanding by linking leadership style, psychological strain, and non-green behavior into a cohesive framework. By establishing that emotional exhaustion mediates the relationship between abusive leadership and environmentally detrimental behaviors, this study introduces a psychologically grounded explanation for how workplace stress can influence sustainability-related actions. This theoretical contribution provides a foundation for future models exploring the indirect mechanisms through which leadership affects discretionary behaviors, extending both COR and JD-R theories into the domain of organizational sustainability and pro-environmental conduct.

## 7. Practical Implications

The findings of this study provide indicative insights for hospitality practitioners and, more broadly, for service industry organizations. While the cross-sectional design limits causal inferences, the results suggest that certain organizational practices may help mitigate the negative effects of abusive leadership and support sustainability-oriented behaviors.

First, the results indicate that reducing abusive leadership behaviors could be beneficial. Organizations may consider leadership development programs focused on emotional intelligence, constructive communication, conflict management, and stress-coping strategies. Such initiatives could potentially support employees’ psychological resources and reduce outcomes like emotional exhaustion and presenteeism, although the effects should be interpreted as suggestive rather than definitive.

Second, the study highlights the potential protective role of co-worker support. Fostering peer networks through mentorship programs, team-building activities, and structured social support opportunities may help employees cope with high job demands and challenging leadership. These supportive practices could indirectly enhance employees’ capacity to engage in discretionary or sustainability-related behaviors.

Third, addressing presenteeism appears to be relevant. Organizations might explore policies that encourage workplace well-being, such as flexible work arrangements, wellness programs, and workload management. While the cross-sectional nature of this study does not allow for firm conclusions about effectiveness, such practices may help reduce strain-related behaviors that could negatively impact employee health and organizational outcomes, including environmental practices.

Finally, the findings suggest that employee well-being and sustainability initiatives are interconnected. Integrating mental health and resilience support with sustainability programs may enhance employees’ resources and engagement in pro-environmental behaviors. These insights emphasize the potential strategic value of aligning human resource practices with environmental responsibility, while acknowledging that further longitudinal or person-centered research is needed to confirm these relationships.

## 8. Limitations and Future Research

Despite its contributions, this study has several limitations that offer avenues for future research. First, the cross-sectional design limits causal inferences; while the results reveal associative relationships between abusive leadership, presenteeism, emotional exhaustion, and non-green behavior, longitudinal or experimental studies are needed to establish the temporal and directional nature of these effects.

Second, the study focuses exclusively on hotel enterprises, which may limit generalizability. Future research should examine other service-oriented or industrial contexts, such as healthcare, retail, or manufacturing, to test whether the observed relationships hold across diverse organizational environments.

Third, although the study collected data from full-time employees in five-star hotels, convenience sampling was used due to logistical constraints and the geographic dispersion of hotels in the Greater Cairo Region. While this approach allowed access to the target population and ensured adequate sample size for PLS-SEM analysis, it limits the generalizability of the findings. Future research could employ probability-based techniques, such as cluster or quota sampling, to enhance external validity and ensure that results are representative of the broader hospitality sector.

Fourth, the reliance on self-reported data may introduce common method bias, despite procedural remedies. Future studies should incorporate multi-source data, including supervisor assessments, peer evaluations, or objective behavioral indicators, to strengthen reliability and validity.

Fifth, although co-worker support was examined as a moderator, other potential contextual or individual factors—such as organizational culture, trust in leadership, employee resilience, or personality traits—may also influence these relationships and warrant exploration.

Sixth, demographic variables (age, gender, work experience) were collected but not included in the structural model, as this was beyond the current study’s scope. Future research could investigate the moderating or control effects of these characteristics to understand individual differences in susceptibility to abusive leadership, emotional exhaustion, presenteeism, and non-green behavior.

Finally, non-green behavior was treated as a general construct. Future studies should examine specific pro-environmental actions, such as recycling, energy conservation, or waste reduction, to provide more granular insights into how workplace stress and leadership behaviors influence sustainability outcomes. This approach can inform more targeted interventions that simultaneously promote employee well-being and organizational sustainability.

## 9. Conclusions

This study provides evidence that abusive leadership is associated with employees’ psychological strain and behavioral outcomes in hotel enterprises. Specifically, abusive leadership was found to be linked with higher levels of emotional exhaustion and presenteeism, which, in turn, were associated with increased non-green behavior. Co-worker support appeared to moderate the relationship between presenteeism and emotional exhaustion, suggesting a potential buffering effect.

Grounded in COR and JD-R theories, the findings offer a framework for understanding how leadership behaviors, resource depletion, and social support may interact to influence employee well-being and environmentally relevant behaviors. The results highlight possible indirect pathways through which leadership behaviors could affect pro-environmental practices, emphasizing the importance of psychological mechanisms in shaping workplace outcomes. These associations warrant further investigation using longitudinal or experimental designs to confirm causal relationships and explore boundary conditions.

From a practical perspective, organizations should:

- Implement structured leadership training to reduce abusive behaviors and enhance emotional intelligence, communication, and conflict management.
- Foster peer support networks, including mentorship programs and team-building initiatives, to buffer workplace stress.
- Develop employee well-being policies, such as flexible work arrangements, workload management, and wellness programs, to minimize presenteeism and resource depletion.

Together, these strategies can enhance employee welfare, sustain discretionary pro-environmental behaviors, and strengthen overall organizational sustainability.

Table 8 below summarizes the key constructs examined in this study, their theoretical foundations, empirical status in prior research, and the gaps addressed by the current work. The table illustrates how abusive leadership influences non-green behavior via emotional exhaustion and presenteeism, while co-worker support serves as a mitigating resource.

## Figures and Tables

**Table 1:** Participant’s profile (N = 400).

		Frequency	Percent
**Gender**	Male	294	78.12
	Female	106	21.88
**Age**	18–<30 years	128	29.26
	30–45 years	178	47.84
	>45	94	22.9
**Education**	High schools	72	19.08
	Bachelor	288	74.81
	Master/PhD	40	6.11

To ensure that participants had sufficient familiarity with their organizational environment, only employees with at least one year of professional experience were included in the study. This approach aligns with Morrison’s (1993) observation that employees generally adapt to their organization’s culture within the first six months of employment.

**Table 2:** Descriptive Statistics of Study Variables.

Construct	Minimum	Maximum	Mean	Std. Deviation	Skewness		Kurtosis	
					Statistic	Std. Error	Statistic	Std. Error
Abusive Leadership (AL)	1.40	4.53	3.3613	0.84143	−0.669	0.122	−0.848	0.243
Presenteeism (PR)	1.00	5.00	3.3354	1.03055	−0.483	0.122	−0.979	0.243
Emotional Exhaustion (EE)	1.00	5.00	3.5764	0.94473	−1.120	0.122	0.378	0.243
Non-green behavior (NGB)	1.00	5.00	2.8045	1.08823	0.109	0.122	−1.193	0.243
Perceived co-worker support (PCWS)	1.00	5.00	3.1800	1.00575	−0.304	0.122	−0.794	0.243

For a 5-point Likert scale, mean scores are interpreted as 1.00–1.80 very low, 1.81–2.60 low, 2.61–3.40 moderate, 3.41–4.20 high, and 4.21–5.00 very high.

**Table 3:** Results of psychometric properties.

Construct	Indicators	Loading	CR	CA	AVE	VIF
**Abusive Leadership (AL)**	AL1	0.767	0.928	0.916	0.517	3.361
	AL2	0.706				
	AL3	0.699				
	AL4	0.642				
	AL5	0.747				
	AL6	0.672				
	AL7	0.735				
	AL8	0.715				
	AL9	0.767				
	AL10	0.742				
	AL11	0.704				
	AL12	0.698				
	AL13	0.723				
	AL14	0.761				
	AL15	0.701				
**Emotional Exhaustion (EE)**	EE 1	0.831	0.935	0.922	0.617	1.850
	EE 2	0.742				
	EE 3	0.800				
	EE 4	0.755				
	EE 5	0.824				
	EE 6	0.781				
	EE 7	0.800				
	EE 8	0.791				
	EE 9	0.741				
**Perceived co-worker support (PCWS)**	PCWS 1	0.790	0.907	0.872	0.661	2.695
	PCWS 2	0.796				
	PCWS 3	0.817				
	PCWS 4	0.838				
	PCWS 5	0.825				
**Presenteeism (PR)**	PR.1	0.851	0.911	0.883	0.632	1.544
	PR.2	0.771				
	PR.3	0.815				
	PR.4	0.738				
	PR.5	0.808				
	PR.6	0.781				
**Non-green behavior (NGB)**	NGB.1	0.860	0.935	0.913	0.742	1.806
	NGB.2	0.833				
	NGB.3	0.888				
	NGB.4	0.870				
	NGB.5	0.853				

**Table 4:** Correlations among latent variables with the square root of AVEs.

Construct	AL	EE	PR	PCWS	NGB
Abusive Leadership (AL)	**0.719**				
Emotional Exhaustion (EE)	0.617	**0.786**			
Presenteeism (PR)	0.371	0.395	**0.795**		
Perceived co-worker support (PCWS)	0.574	0.436	0.312	**0.813**	
Non-green behavior (NGB)	0.484	0.427	0.568	0.444	**0.861**

Note: Bold ratios show the square root of AVE.

**Table 5:** Discriminant validity (HTMT).

Construct	AL	EE	PR	PCWS	NGB
Abusive Leadership (AL)					
Emotional Exhaustion (EE)	0.673				
Presenteeism (PR)	0.408	0.437			
Perceived co-worker support (PCWS)	0.656	0.488	0.355		
Non-green behavior (NGB)	0.529	0.466	0.635	0.498	

**Table 6:** Direct and moderation effects.

H	Structural Paths	Path Coefficient (*β*)	*p*-Values	Effect Size (f^2^)	Result
***Direct Effect***					
H1	AL → EE	0.35	<0.01	0.217	Supported
H2	AL → PR	0.38	<0.01	0.144	Supported
H3	PR → EE	0.18	<0.01	0.082	Supported
H5	PCWS → EE	−0.24	<0.01	0.146	Supported
H7	AL → NGB	0.38	<0.01	0.190	Supported
H8	EE → NGB	0.19	<0.01	0.086	Supported
***Moderating Effect***					
H6	PR × PCWS → EE	−0.18	<0.01	0.073	Supported

PR R^2:^ = 0.14, EE R^2:^ = 0.23, NGB R^2:^ = 0.28.

**Table 7:** Mediation analysis’ Bootstrapped Confidence Interval.

Hypothesis		Path a	Path b	Indirect Effect	SE	t-Value	Bootstrapped Confidence Interval		Mediation
							*95% LL*	*95% UL*	
H4	AL → PR → EE	0.380	0.180	0.068	0.032	2.138	0.006	0.131	Yes
H9	AL → EE → NGB	0.350	0.190	0.067	0.029	2.293	0.010	0.123	Yes

**Table 8:** Summary of key findings and implications of the study.

Construct/Concept	Key Findings/Insights	Implications from Current Study
Abusive Leadership	Confirmed as a significant predictor of emotional exhaustion and presenteeism.	Highlights the latent threat to hotel sustainability and employee well-being; interventions needed to reduce abusive supervisory behaviors.
Emotional Exhaustion	Mediated the effect of abusive leadership on non-green behavior (Cropanzano et al., 2003; N. Yang, 2023).	Demonstrates that psychological strain limits employees’ discretionary environmental behaviors; reducing burnout can enhance green practices.
Presenteeism	Acted as a mediator linking abusive leadership to emotional exhaustion and non-green behavior (Biron et al., 2006; Khairy, 2020).	Suggests managing attendance pressures and health resources is crucial for sustaining environmental compliance.
Non-Green Behavior	Increased under abusive leadership via emotional exhaustion and presenteeism.	Indicates leadership interventions are essential not only for service quality but also to protect sustainability outcomes.
Co-Worker Support	Moderated the relationship between abusive leadership and emotional exhaustion/non-green behavior (H. Wang & Tang, 2022).	Emphasizes fostering peer support as a buffer to preserve employee well-being and green behavior even under negative leadership.
Underlying Theories	Provided explanatory framework for mediating and moderating effects.	Confirms relevance of resource-based perspectives in understanding environmental behavior under stressful leadership.

## Data Availability

Data are available upon request from researchers who meet the eligibility criteria. Kindly contact the first author privately through e-mail.

## Appendix A

**Table A1:** Model fit and quality indices (Kock, 2021).

	Assessment	Criterion	Mark
Average path coefficient (APC)	0.270, *p* < 0.001	*p* < 0.05	√
Average R-squared (ARS)	0.216, *p* < 0.001	*p* < 0.05	√
Average adjusted R-squared (AARS)	0.211, *p* < 0.001	*p* < 0.05	√
Average block VIF (AVIF)	1.808	acceptable if ≤ 5, ideally ≤ 3.3	√
Average full collinearity VIF (AFVIF)	2.071	acceptable if ≤ 5, ideally ≤ 3.3	√
Tenenhaus GoF (GoF)	0.385	small ≥ 0.1, medium ≥ 0.25, large ≥ 0.36	√
Sympson’s paradox ratio (SPR)	0.857	acceptable if ≥ 0.7, ideally = 1	√
R-squared contribution ratio (RSCR)	0.845	acceptable if ≥ 0.9, ideally = 1	√
Statistical suppression ratio (SSR)	1.000	acceptable if ≥ 0.7	√
Nonlinear bivariate causality direction ratio (NLBCDR)	1.000	acceptable if ≥ 0.7	√
Standardized root mean squared residual (SRMR)	0.086	acceptable if ≤ 0.1	√
Standardized mean absolute residual (SMAR)	0.068	acceptable if ≤ 0.1	√
Standardized chi-squared with 779 degrees of freedom (SChS)	20.252, *p* < 0.001	*p* < 0.05	√
Standardized threshold difference count ratio (STDCR)	0.979	acceptable if ≥ 0.7, ideally = 1	√
Standardized threshold difference sum ratio (STDSR)	0.928	acceptable if ≥ 0.7, ideally = 1	√
